# A Multi-User Personal Indoor Localization System Employing Graph-Based Optimization

**DOI:** 10.3390/s19010157

**Published:** 2019-01-04

**Authors:** Michał R. Nowicki, Piotr Skrzypczyński

**Affiliations:** Institute of Control, Robotics and Information Engineering, Poznan University of Technology, 60-965 Poznan, Poland; piotr.skrzypczynski@put.poznan.pl

**Keywords:** indoor positioning, graph-based optimization, WiFi fingerprinting

## Abstract

Personal indoor localization with smartphones is a well-researched area, with a number of approaches solving the problem separately for individual users. Most commonly, a particle filter is used to fuse information from dead reckoning and WiFi or Bluetooth adapters to provide an accurate location of the person holding a smartphone. Unfortunately, the existing solutions largely ignore the gains that emerge when a single localization system estimates locations of multiple users in the same environment. Approaches based on filtration maintain only estimates of the current poses of the users, marginalizing the historical data. Therefore, it is difficult to fuse data from multiple individual trajectories that are usually not perfectly synchronized in time. We propose a system that fuses the information from WiFi and dead reckoning employing the graph-based optimization, which is widely applied in robotics. The presented system can be used for localization of a single user, but the improvement is especially visible when this approach is extended to a multi-user scenario. The article presents a number of experiments performed with a smartphone inside an office building. These experiments demonstrate that graph-based optimization can be used as an efficient fusion mechanism to obtain accurate trajectory estimates both in the case of a single user and in a multi-user indoor localization system. The code of our system together with recorded dataset will be made available when the paper gets published.

## 1. Introduction

The demand for accurate indoor positioning services has grown significantly in the last decade, resulting in an increasing interest in localization methods that work in environments where GPS (Global Positioning System) signal is unreliable or unavailable at all.

The application areas that may benefit from reliable personal localization in GPS-denied environments are numerous. However, the largest interest in indoor positioning is generated by applications related to new business and advertising opportunities. Location-aware advertising and the possibility to analyze the traffic patterns of consumers make the indoor localization services attractive to owners or managers of many commercial buildings. This trend is confirmed by a market report [1] that predicts very fast growth of the indoor positioning systems market.

Among the approaches to indoor positioning, the most interesting are those that are infrastructure-free: they do not need to deploy any dedicated active beacons (e.g., iBeacons [2]) or artificial landmarks in the environment, which reduces the costs of installation and maintenance of a localization system. On the other hand, to make the localization services available to a large number of potential users, smartphones and other mobile computing devices like tablets are widely used as the sensing component of indoor positioning systems [3].

Systems utilizing camera images [4,5], Bluetooth Low Energy [6], anomalies of the ambient magnetic field [7], and even light sources [8] have been proposed for localization with smartphones. A brief survey of the indoor positioning techniques focusing on the suitability of different sensing modalities is presented in Ref. [9]. However, the most widely used localization modality for personal indoor positioning is the WiFi signal. Nowadays, WiFi networks are ubiquitous in modern buildings making WiFi-based positioning affordable and relatively easy to deploy on site. In recent years, a number of companies attempted to commercialize their localization solutions. In specific sites commercial mobile location services are provided by Skyhook (Skyhook website: https://www.skyhook.com/), which offers hybrid positioning technology involving WiFi, GPS, cell towers, and IP addresses. More recently, the Indoo.rs company (Indoo.rs website: https://indoo.rs/) offered a solution that mixes the iBeacons technology and WiFi-based positioning for indoor localization, deploying their system in several large public sites. However, this system and other similar solutions require dense wireless signal maps [10], that are laborious to prepare and maintain.

Therefore, we focus on WiFi-based positioning algorithms that do not need to survey the entire environment the localization system is deployed in. The ability to localize with a limited number of known WiFi fingerprints can be achieved by employing graph-based optimization to relax a network of constraints stemming from the matches between the known fingerprints and the consecutive scans perceived along the user’s trajectory. Constraints imposed by the motion of the user, estimated by a Personal Dead Reckoning (PDR) system on the smartphone are used to join the consecutive poses into a trajectory. A single-user indoor positioning system based on this approach has been presented in our paper [11].

In this journal article we go beyond the previous approach, making a number of original contributions over our earlier research and the state-of-the-art:

- We re-formulate the graph-based optimization approach for PDR and WiFi constraints to jointly optimize the trajectories of multiple users in the same building, improving the accuracy of the estimated position for involved users.
- We introduce the hyperedges that efficiently represent in the graph multiple constraints imposed on the agent pose by positioning with the Weighted *k*-Nearest Neighbor technique.
- We show how the user’s step length, which is a key parameter in the PDR, can be jointly estimated in the graph-based formulation during the localization.
- We demonstrate that with the presented approach it is possible to update the existing WiFi map.

The code of our system will be publicly available at GitHub (https://github.com/LRMPUT/IndoorGraphLocalization) with the final version of the paper.

## 2. Related Work

In this research we are interested in infrastructure-free, smartphone-based indoor positioning exploring the WiFi signals from the access points already available in the environment and the proprioceptive sensors that are present in almost every modern mobile device: accelerometers, gyroscopes, and magnetometers.

Indoor positioning using WiFi signals is investigated for more than a decade. A survey of classic approaches can be found in Ref. [12]. If positions of the access points with respect to the floor plan are known, then WiFi signal triangulation can be used. Three or more line-of-sight AP positions are necessary to determine the location of the WiFi adapter using the distance to each AP measured from the parameters of the received signal [13]. Unfortunately, direct use of the received signals is complicated indoors, because walls, furniture, and other objects located in the environment, including the people, attenuate or scatter the WiFi signals in a way that is hard to model and predict [14]. A solution to estimate the user position with respect to the WiFi APs using a signal propagation model was proposed in Ref. [15]. This approach using also inertial data and employing Kalman filtering is computationally efficient and runs on a smartphone, but in real indoor environments it yields inaccurate results due to the simplified signal propagation model. Although it is possible to build a map of WiFi signal strength using Gaussian processes [16], thus avoiding to model explicitly the propagation of signals, this method is computation intensive and requires a laborious survey of the whole area under consideration.

Conversely, approaches employing WiFi fingerprinting do not need to model the signal propagation at all and do not need to determine exact positions of the APs, or even to know the number of the APs deployed in the area. This is of great practical importance, as in many environments not only a location of objects altering the WiFi signals changes frequently, but some wireless networks appear or disappear at random, because routers, printers, and other devices are often moved between locations.

Currently, the most widely applied fingerprinting-based positioning technique is Weighted *k*-Nearest Neighbor (WKNN) [17]. This relatively simple method selects *k* most similar fingerprints from the pre-surveyed database describing the environment and computes the user position as a weighted average of the positions of these fingerprints. Weights are related to the similarity between the matched fingerprints. A drawback of this technique is the need to manually tune its parameters for the given environment. Although attempts to develop WiFi-based indoor positioning algorithms that require a minimum effort at the design stage, for example by employing end-to-end machine learning [18], have been presented in the literature, the achieved localization accuracy is not much improved with respect to WKNN.

In general, the need for a database of densely distributed scans is a common drawback of positioning algorithms using only the WiFi fingerprints. However, in practical applications, we rarely are facing the problem of global localization with WiFi data, without any prior information about the state of the agent. In personal localization with smartphones, a prediction of the user’s current pose giving the previous pose on the path can be achieved by means of PDR, exploiting the inertial sensors of the mobile device. Combining WiFi fingerprinting with PDR pose estimates makes it possible to employ filtering algorithms to track the position of the user. The Extended Kalman Filter (EKF) [19] allows the positioning system to run on a device with limited computing resources. Unfortunately, the EKF algorithm is prone to linearization errors on the highly uncertain measurements from a smartphone and does not support non-Gaussian user position hypotheses, which can emerge due to the ambiguity in WiFi scan matching. The most popular alternative approach is Particle Filter (PF), which can track multiple hypotheses of the user position [20], and enables to easily incorporate the user position constraints defined by the known environment map [21]. Recently, an indoor localization system employing an advanced version of PF was successfully demonstrated in a challenging scenario of a museum site in a large historic building [22]. On the other hand, PF often requires a large number of particles to converge to the true position and is computation intensive, which makes it less suitable for implementation on a smartphone [23]. The performance of EKF and PF in the context of indoor positioning is compared in Ref. [24].

Another prominent approach borrowed from the area of mobile robotics is graph-based localization. Nowadays, solving the Simultaneous Localization and Mapping (SLAM) problem transformed into a graph of constraints by means of computationally efficient non-linear optimization is considered the state-of-the-art in robotics [25]. Graphs are convenient data structures to encode topological relations, hence a number of indoor positioning systems employ graphs to represent the indoor space or the possible user paths. Hilsenbeck et al. [26] use a graph structure to achieve an adaptive quantization of the indoor space that in turn reduces the state-space dimensionality in a particle filter-based localization algorithm that combines PDR and WiFi signal strength measurements. This results in a substantially reduced number of particles in the filter and improved performance on a smartphone. Available walking paths abstracted from a floor plan are represented as a graph in Ref. [27]. This approach assumes that users move only along edges in the graph that form Markov chains. The limited number of possible motions encoded by Markov chains makes it possible to improve the performance of Kalman filter used to fuse WiFi fingerprinting and inertial measurements of the user’s pose. Graphs used just to represent the environment’s topology do not encode the constraints related to measurements, and therefore cannot represent the entire localization problem, like the graphs used in SLAM do. Although graph optimization in SLAM is usually applied in the context of range-based, visual or inertial-visual sensing, it has been demonstrated [28] that the idea of graph optimization can be used to integrate information obtained with several sensing modalities in a plug-and-play manner. Signal strength from WiFi APs was also used as the sole source of localization information in an off-line SLAM system employing graph optimization [29]. Whereas none of these publications tackled the problem of modelling WiFi fingerprinting constraints in a graph-optimization framework, our previous research [11] demonstrated the feasibility of the so-called factor graphs [30] for modelling and integrating the constraints imposed by WiFi and PDR in the indoor positioning problem. Assuming Gaussian sensor noise, factor graphs provide an efficient way to represent large, non-linear least-squares optimization problems, which are typical in robotic SLAM, but are present also whenever a large number of WiFi fingerprints and user motion estimates have to be fused into a coherent representation. We have demonstrated the real-time performance of this approach on a typical smartphone employing a general-purpose graph-optimization software framework (library) commonly used in robotics. Very recently, this system was extended by adding non-metric constraints [31], which improved robustness and allowed to handle multi-floor environments. Among the indoor positioning systems described in the literature, the approach most similar to our system is the FastGraph [32]. Although this work has been published very recently, it does not take advantage of the existing graph-optimization packages, but constructs a graph of constraints that explicitly consider the WiFi signal propagation using the Log-Distance Path Loss Model. This graph is then relaxed to a minimum energy state using a force-directed approach following the spring analogy for edges-constraints. Comparing it to our approach, FastGraph in its default configuration employs dedicated devices to collect WiFi fingerprints, whereas we need only smartphones/tablets held by the users, and requires manually setting a number of values that govern the relaxation process. Our probabilistic solution requires only to define sensor models that determine the behavior of constraints.

Even though the PF and graph-based localization methods offer satisfying accuracy and robustness, major obstacles that hinder the wider acceptance of WiFi-based indoor positioning in commercial applications are the time and effort necessary to build and maintain a database of fingerprints. An example of a large database of WiFi data is given in Ref. [33]. It was collected by volunteer users, locating the WiFi scans upon a manual input on the known map. Although the volunteers could roughly estimate their position using a variant of the WKNN approach, the process of collecting the data was laborious, and it is one of the few data sets of this kind that are available. Hence, approaches that do not need such a database, or can collect it automatically are of great practical importance [34]. Automatization of the site survey by using another localization system, e.g., RGB-D SLAM [35], requires additional equipment and is rather impractical for large environments such as shopping malls. Thus, approaches such as UnLoc [36] started to emerge. UnLoc is a smartphone-based positioning system that exploits the possibility to track mobile devices of multiple users by means of dead reckoning and landmarks, in order to collect the database of WiFi signals without any explicit involvement of these users. A similar idea was implemented in Ref. [21] using particle filtering and leveraging the role of a known map of the environment. Laoudias et al. [20] used the same general idea, termed crowdsourcing, to avoid the need to survey the site, and an explicit participation of the users of diverse mobile devices in collecting the WiFi signals. The WicLoc system [37] employed the WKNN algorithm to assign weights to APs and to achieve room-level localization from crowdsourced data. Most of the crowdsourcing methods employ point-based maps of the fingerprints, where the WiFi scans are pinpointed to locations distributed in a regular grid or along predefined paths. A different approach is taken in Ref. [38]. The map of WiFi fingerprints is abstracted by the semantics graph, with possible paths of the users as edges, and specific locations as vertices. Smartphone-based dead reckoning and user activity detection are employed to locate the trajectories and construct a map of fingerprints.

Although crowdsourcing methods become increasingly popular for indoor positioning with smartphones, the approaches proposed in this area do not take advantage of the existing body of research in multi-robot localization and SLAM. A recent survey on multi-robot SLAM [39] suggests that approaches based on graph optimization (i.e., factor graphs) efficiently handle constraints between the agents when they observe the same environment, which is essential to localize multiple users and build the map simultaneously with a minimal involvement of these users.

## 3. Graph-Based Optimization

In this research, we adopt an approach to SLAM commonly used in robotics to the problem of indoor positioning with WiFi fingerprints and inertial estimation of the user motion. In the last decade, non-linear optimization algorithms started to be applied to the SLAM problem [25]. In this formulation of SLAM, the maximum-a-posteriori (MAP) estimate of the unknown variables is computed given a set of measurements, that can be expressed as a function of the agent state. This approach avoids large linearization errors and approximations made by the SLAM algorithms employing filtration, thus yielding accurate estimates of the agent state [40]. A full probabilistic formulation of SLAM as a Bayesian filtering problem with explanation of the essential assumptions as to the Markovian nature of the observed processes and statistical independence of variables can be found in the vast body of literature on robot localization, e.g., in our survey paper [41]. However, for the sake of clarity, we provide here a brief introduction to the factor graph formulation of SLAM, focusing on the physical meaning of particular components. Later, we use this formulation to comment the changes that were necessary to adopt this framework for indoor positioning.

The SLAM problem is cast as estimating the probability distribution of the state vector x, given the measurements vector z. If the measurement errors follow a zero mean normal distribution, then the likelihood of the measurements is Gaussian. The distribution over the states given the measurements is proportional to the likelihood of the measurements given the states:

(1) $$p\left(z|x\right)\propto \exp\left({\left(\hat{z}-z\right)}^{T}\Omega \left(\hat{z}-z\right)\right),$$

where $\hat{z}$ is the prediction of the measurements, given the state, and Ω is the information matrix of the conditional measurements. To solve the problem we need to compute a state $x^{*}$ that maximizes (1):

(2) $$x^{*}=\underset{x}{\mathrm{argmin}}\sum_{m=1}^{M}{\left(h_{m}\left(x\right)-z_{m}\right)}^{T}\Omega_{m}\left(h_{m}\left(x\right)-z_{m}\right),$$

where assuming the total number of *M* measurements, $h_{m}\left(x\right)={\hat{z}}_{m}$ yields the *m*-th predicted measurement/observation applying the sensor model. Defining the difference between the *m*-th observation and its prediction as the error function:

(3) $$e_{m}\left(x\right)=h_{m}\left(x\right)-z_{m},$$

we can define the minimized function in Equation (2) as:

(4) $$F\left(x\right)=\sum_{m=1}^{M}e_{m}{\left(x\right)}^{T}\Omega_{m}e_{m}\left(x\right).$$

Using the Taylor approximation (the sensor model is a non-linear function of the state), we re-write (4) applying small increments Δx and the linearization point $\overset{˘}{x}$ in the state space:

(5) $$F\left(\overset{˘}{x},\Delta x\right)\approx \Delta x^{T}\sum_{m=1}^{M}H_{m}\Delta x+2\sum_{m=1}^{M}b_{m}\Delta x+\sum_{m=1}^{M}e_{m}{\left(x\right)}^{T}\Omega_{m}e_{m}\left(x\right),$$

where $H_{m}=J_{m}^{T}\Omega_{m}J_{m}$ with the Jacobians $J_{m}={\left.\frac{\partial h_{m}\left(x\right)}{\partial x}\right|}_{x=\overset{˘}{x}}$. Then, it is easy to show that the $\Delta x^{*}$ minimizing the quadratic form in Equation (5) can be found solving the equation:

(6) $$H\Delta x^{*}=b,$$

where the Hessian is defined as $H=\sum_{m=1}^{M}H_{m}$, and $b=\sum_{m=1}^{M}b_{m}$. Due to the non-linearity of the measurement model we need to apply a non-linear numerical method to solve (6), i.e., Gauss-Newton or Levenberg-Marquardt algorithm. In particular, the solution can be computed efficiently modelling the problem as a factor graph. Once proper error functions with sensor models and their Jacobians are defined, the state vector minimizing (6) can be computed by one of the available generic factor graph-optimization packages applying a very efficient numerical solver [25].

**Figure 1:**
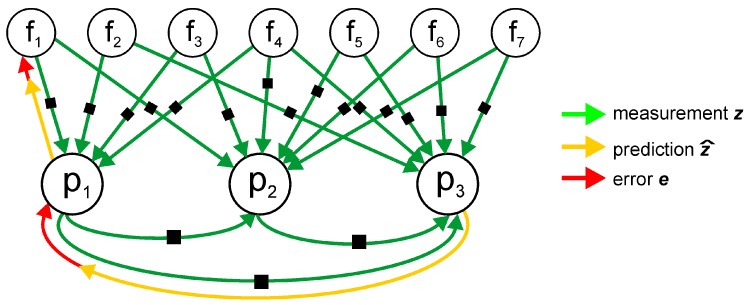
Factor graph representation of the Simultaneous Localization and Mapping problem with agent poses and generalized landmarks. The factors shown as black rectangles relate either pairs of agent poses (motion estimates) or poses and landmarks (external measurements). Notice that factors are not shown on further graphs explicitly for the sake of clarity of the figures.

In the case of indoor positioning, the instantaneous agent state is just its 2-D pose (*x*, *y* position and heading angle θ) in the environment, while the unknown state variables x are representing the set of past poses (i.e., agent trajectory) and the environment map, which is represented as a discrete set of landmarks that can be observed by the agent. The dependencies between the poses of the agent, the detected landmarks, and the measurements can be conveniently represented as a factor graph (Figure 1), whose vertices are the poses of the agent and the landmarks, and the edges represent constraints imposed by the motion of the agent, and measurements of the landmark positions, The measurements are represented in the graph as factors, shown in Figure 1 by small black rectangles.

In our system the factor graph has two kinds of vertices: agent poses are represented by p, while f represents landmarks, thus the state is formally defined as $x={\left[p,f\right]}^{T}$. The edges represent either the constraints ${\left[\theta ,t\right]}_{i}^{T}\in \mathrm{SE}(2)$ imposed by agent motion between the (i−1)-th and *i*-th pose, or the constraints $t_{ij}\in R^{2}$, stemming from the measurements between the *i*-th pose of the agent, and the *j*-th landmark. Whenever *k* landmarks are measured from the same pose of the user at the same time a “hyperedge” can appear in the graph that represents all the *k* constraints $t_{i,j}\in R^{2},$ for j=1…k imposed by the simultaneous measurements. The uncertainty of constraints imposed by landmark observations is represented by their information matrices $\Omega_{f}$ computed upon the empirical model of measurements, while the uncertainty of motion constraints $\Omega_{p}$ depends on the accuracy of the agent ego-motion estimation. Considering single landmark-to-pose constraints as hyperedges with *k* = 1 within the introduced graph structure, the function to minimize in the localization problem is given as:

(7) $$x^{*}=\underset{p,f}{\mathrm{argmin}}\left(\sum_{i=1}^{n}e_{f(i)}^{T}\Omega_{f}e_{f(i)}+\sum_{i=2}^{n}e_{p(i)}^{T}\Omega_{p}e_{p(i)}\right),$$

where *n* is the number of recorded agent poses, $e_{f(i)}=e\left(p_{i},f_{1},\ldots ,f_{k},t_{i,1},\ldots ,t_{i,k}\right)$ and $e_{p(i)}=e\left(p_{i-1},p_{i},{\left[\theta ,t\right]}_{i-1,i}^{T}\right)$ are error functions for the landmark-to-pose and pose-to-pose constraints, respectively.

Some factors can represent loop closures, which are constraints between poses representing the locations that are re-visited by the agent. These constraints are particularly important, as they inject new information to the graph, thus enabling the optimization algorithm to reduce the agent trajectory drift. Optimization reduces the drift only if at least one vertex in the factor graph has at least two incoming edges. This implies the existence of constraints other than those produced by agent motion along the trajectory. It should be noticed, that constrains from wrong loop closures or spurious landmark measurements have a negative influence on the optimization process because of the least-squares formulation of the estimation problem represented by the factor graph [42].

This framework is often implemented by a system divided into two parts: the front-end, that abstracts sensory measurements into the graph-based representation that is amenable for optimization, and the back-end, that employs a graph-optimization library to solve (7). One of the most widely used factor graph-optimization packages is the g${}^{2}$o [43], employed in a number of robotic SLAM systems. This library is also used in our system and is configured to apply the Preconditioned Conjugate Gradient algorithm (PCG), which is fast but requires a good initial guess of the location of the graph vertices. This choice is a trade-off between robustness and computation speed. When using the PCG, the memory consumption grows linearly in the number of state variables. Although in theory factor graph optimization has cubic complexity in the number of state variables, the actual complexity is much lower due to the sparse nature of the SLAM problem. Following the argumentation given by Strasdat et al. [40] we consider three operations that cause the computation burden: evaluation of the error functions and Jacobians, building the linear system, and solving the linear system. The first operation is linear in the number of observations, thus the worst-case complexity can be estimated as O(nm) for *n* agent poses and *m* measurements, assuming that all fingerprints are seen from all poses, while in practice only a small fraction of the fingerprints is detectable from each pose. The second operation is quadratic in *n* and linear in *m*, while the third one is cubic in *n*. Because in the indoor positioning task the number of poses is relatively small, while the solvers available in g${}^{2}$o efficiently exploit sparsity of Hessian in the linear system [43], the cost of computations is dominated by evaluating the Jacobians and building the linear system. Hence, the computational complexity can be estimated rather as $O(n^{2}m)$.

Adopting the SLAM framework to indoor positioning with WiFi data requires to re-define the notion of landmarks, as in our formulation the “landmarks” represent the locations of WiFi fingerprints. The pose-to-pose constraints, that in mobile robotics are usually established using odometry (either wheeled or visual), are yielded by PDR in the case of our smartphone-based personal localization system. In turn, the use of both, WiFi scans and PDR, requires proper definitions of the error functions $e_{f(i)}$ and $e_{p(i)}$, respectively. These functions should account for the uncertainty characteristics that are specific to the measurements taken with the limited accuracy sensors of a smartphone [11].

## 4. Constraints in the Graph-Based Optimization

**Figure 2:**
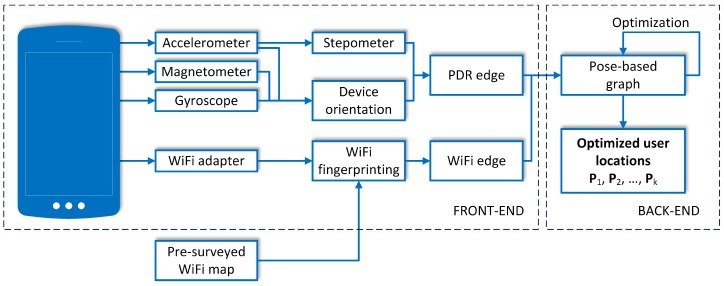
Overview of the processing performed on a mobile device to obtain personal localization.

An overview of the processing blocks in the single-user version of the proposed positioning system is presented in Figure 2. The front-end is composed of blocks that process data from the sensors available in a mobile device to implement the PDR for user motion estimation, and WiFi-based localization employing WKNN. The measurements processed by these blocks are then represented as edges in the g${}^{2}$o-based back-end that provides the best pose guess after the optimization procedure. In this version the whole system is self-contained and can be implemented on a smartphone, running in real-time, as demonstrated in Ref. [11]. The following subsections present the used components in more details.

### 4.1. Pedestrian Dead Reckoning

Dead reckoning for pedestrians involves two components: a method that estimates the distance covered by the person, and a method that estimates the change in orientation of this person with respect to the inertial frame. A combination of these estimates makes it possible to roughly estimate the position and orientation of the user in 2-D, assuming that he/she walks on the flat floor. Both components can be implemented using the inertial sensors of a mobile device: accelerometers, and gyroscopes. If a magnetometer is available in the device, then its absolute heading measurements may be integrated as well.

The first component of PDR, often called stepometer, can be implemented detecting and counting individual steps made by the person. We employ the Fast Fourier Transform (FFT) of the accelerometer signal in a moving window of samples to detect the steps, as proposed in Ref. [44]. If the dominant frequency *f* detected by FFT is between 1.3 and 2.2 Hz, then the steps are considered to be detected. The distance covered in the *i*-th iteration is computed using the formula:

(8) $$d_{i}=l_{s}\cdot f_{i}\cdot \frac{n}{n_{w}},$$

where $f_{i}$ is the dominant frequency in the moving window of *i*-th iteration, *n* is the number of accelerometer measurements since the last distance computation, and $n_{w}$ is the length of the moving window. The default step length $l_{s}$ is estimated to be equal to 0.65 m for an adult male person [11]. In the implemented PDR $n_{w}$ is equal to 256 measurements, which made it possible to detect sudden movements of the person, while providing satisfactory accuracy of step recognition when the accelerometer provides 200 measurements per second. Although a longer window could result in a more accurate estimation of the dominant frequency, it would have an adverse effect on the detection of the start and stop of the motion. On the other hand, a much shorter window yields more false positives in step detection.

The second component of the PDR is the algorithm that estimates the heading angle of the device, thus allowing the PDR to use the estimated distance to compute the position with respect to the external coordinates. The orientation of the mobile device is computed fusing inputs from the accelerometers, magnetometer, and gyroscope. Those sensors are present in most of the modern smartphones and tablets. Unfortunately, low-cost sensors are often used in smartphones and tablets that results in degraded accuracy of the measurements. Hence, we apply an Adaptive Extended Kalman Filter (AEKF) algorithm to compute the heading estimate. This algorithm, presented in detail in Ref. [45], combines the magnetometer, gyroscope and accelerometer readouts to estimate the smartphone coordinate system in the ENU (East, North, Up) inertial coordinates. Our AEKF uses a quaternion to represent the orientation and estimates three gyroscope biases. The adaptation mechanism detects the type of motion from the accelerometers data, distinguishing between smooth motion and rapid movements of high acceleration. Then, the covariance matrices used in the Kalman filter are adjusted according to the motion type, resulting in reliable heading estimation in a wide range of operational conditions. Although we are aware of more sophisticated PDR methods published recently [46], we use the approach introduced in Ref. [11], only slightly modified for this research, as we found it accurate and reliable enough, while it is simple and does not consume many resources of the smartphone.

**Figure 3:**
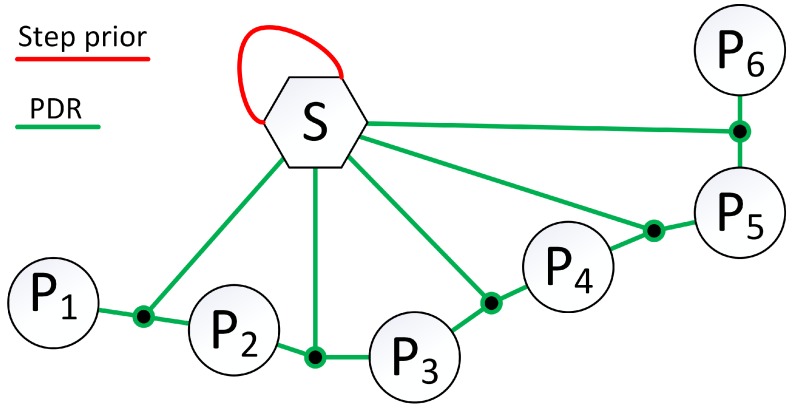
Each PDR edge connects two poses and node containing step length estimate. An edge representing step length prior is added to the step length node to constrain estimates to plausible values.

The graph-based formulation of the PDR edge is different in the current version in comparison to our previous work [31]. Firstly, we assume that the user walked with the heading angle computed as the average of the heading angles of the two poses joined by that edge, while previously we assumed that the heading angle is the orientation of the original pose with half of the measured angle change. We believe the current formulation is more suited in the cases when the orientation change is less accurate. Secondly, the step length for each user is assumed to be a parameter specific to this person, and can be estimated during the positioning system operation. Due to this change, the system can adapt to different users, thus obtaining more accurate pose estimates. With this information it is possible to define the error of the PDR graph edge joining ((i−1)-th) and *i*-th pose as:

(9) $$e_{p(i)}=\left[\begin{array}{c} x_{i}-x_{i-1}-l_{s}\cdot f_{i}\cdot \cos\left(\frac{\theta_{i-1}+\theta_{i}}{2}\right)\\ y_{i}-y_{i-1}-l_{s}\cdot f_{i}\cdot \sin\left(\frac{\theta_{i-1}+\theta_{i}}{2}\right)\\ \left(\theta_{i}-\theta_{i-1}\right)-\Delta \theta_{i-1,i} \end{array}\right],$$

where $\left(x_{i},y_{i},\theta_{i}\right)$ are coordinates of the *i*-th user pose, $l_{s}$ is the estimated step length, $f_{i}$ is the measured dominant frequency of walking, and $\Delta \theta_{i-1,i}$ is the measured change in the heading angle. The exemplary graph with PDR edges is presented in Figure 3.

The graph-based optimization utilizes iterative optimization procedures, and a good initial guess of the node location is necessary to achieve satisfactory results. Therefore, a new, *i*-th user pose is initialized with the (*i*−1)-th user location incremented by the PDR measurement. We also try to keep the graph optimized at all times to reduce the possibility of finding suboptimal local minimum of the cost function. The iterative procedures also require the Jacobians to determine the direction of increments that minimizes the error. In the case of the PDR, the corresponding Jacobian $J_{\mathrm{pdr}}=\left[\begin{array}{ccc} \frac{\partial J_{\mathrm{pdr}}}{\partial p_{i-1}}, & \frac{\partial J_{\mathrm{pdr}}}{\partial p_{i}}, & \frac{\partial J_{\mathrm{pdr}}}{\partial l_{s}} \end{array}\right]$ has to be computed with respect to the (i−1)-th user location $p_{i-1}$, *i*-th user location $p_{i}$ and estimated step length $l_{s}$. The Jacobian matrix has a size of 3×7 and is analytically computed, which speeds up the optimization process, compared to numerical approximations of these Jacobians:

(10) $$J_{\mathrm{pdr}}=\left[\begin{array}{ccccccc} -1 & 0 & 0.5\cdot l_{s}\cdot f_{i}\cdot \sin\left(\frac{\theta_{i-1}+\theta_{i}}{2}\right) & 1 & 0 & 0.5\cdot l_{s}\cdot f_{i}\cdot \sin\left(\frac{\theta_{i-1}+\theta_{i}}{2}\right) & -f_{i}\cdot \cos\left(\frac{\theta_{i-1}+\theta_{i}}{2}\right)\\ 0 & -1 & -0.5\cdot l_{s}\cdot f_{i}\cdot \cos\left(\frac{\theta_{i-1}+\theta_{i}}{2}\right) & 0 & 1 & -0.5\cdot l_{s}\cdot f_{i}\cdot \cos\left(\frac{\theta_{i-1}+\theta_{i}}{2}\right) & -f_{i}\cdot \sin\left(\frac{\theta_{i-1}+\theta_{i}}{2}\right)\\ 0 & 0 & -1 & 0 & 0 & 1 & 0 \end{array}\right].$$

As the error of the graph edge for the PDR measurement contains errors in the different units, the metric error of the position and the heading error of the orientation must be properly weighted by the information matrix according to the uncertainty of the measurements. These uncertainties could be determined by the calibration procedures individually for each mobile device, which is impractical for real-life applications. Therefore, we assume that the real uncertainty can be approximated with a simplified information matrix:

(11) $$\Omega_{p}=\left[\begin{array}{ccc} k_{p} & 0 & 0\\ 0 & k_{p} & 0\\ 0 & 0 & k_{o} \end{array}\right],$$

with parameter $k_{p}$ defining the uncertainty of the position and $k_{o}$ being responsible for the uncertainty of the orientation. In the proposed system, $k_{p}$ is set to 1, and $k_{o}$ is set to 15 following our experiments from Ref. [11].

During the optimization, the parameter $l_{s}$ is estimated to determine the user-specific step length. In the beginning, it was assumed that the step length is equal to 65 cm ($s_{0}=0.65$) but the value might change without constraints. In order to limit the parameter values to sensible estimates, a unary graph edge is added to the graph node constraining $l_{s}$ value with the error defined as:

(12) $$e_{s}=\left[\begin{array}{c} l_{s}-l_{s_{0}} \end{array}\right],$$

where $l_{s_{0}}$ is the assumed prior step length, and is equal to 0.65 m in our system. This prior constraint has a corresponding information matrix $\Omega_{s}=k_{s}$. The parameter $k_{s}$ determines the uncertainty of the estimated step length and could be based on the typical distribution of the step lengths of the population if such information would be known. In the proposed system, the parameter $k_{s}$ is set to 200 to prevent the system from finding improbable step lengths that result in optimization more likely to change the location of the user poses than on introducing changes to step length estimate. Despite high confidence on the step length prior, the system is still capable of step length estimation when all information suggests that the user’s step length is shorter or longer than anticipated. From the formal point of view, an additional term defined by (12) and the information matrix $\Omega_{s}$ is added to the optimization problem formulated by (7), as the third type of constraint.

### 4.2. WiFi Fingerprinting

An absolute position of the user in the global coordinates can be determined on the basis of a single WiFi scan, by matching this scan to other WiFi fingerprints captured in known locations. Those fingerprints can be stored in a pre-surveyed database or they can be scans obtained previously along similar trajectories by the same user or by others.

To compute the absolute location from the matching of fingerprints we employ the WKNN approach [47], which proved to be reliable in a number of other positioning systems [17,27]. At first, WKNN finds the *k* most similar scans applying the chosen similarity norm *s*. We consider matches between scans only if at least shared_threshold of WiFi networks are found in both of scans. Then, the user position p, related to the recently obtained WiFi scan X is determined as a weighted average of the positions of these *k* scans:

(13) $$p=\frac{\sum_{i=1}^{k}s\left(X,Y_{i}\right)f_{i}}{\sum_{i=1}^{k}s\left(X,Y_{i}\right)},$$

where $\left(Y_{1},\ldots ,Y_{k}\right)$ are the *k* most similar scans, $f_{i}=\left(x_{i},y_{i}\right)$, i=1,2,…,k, are positions of these scans (i.e., our “landmarks”).Because the structure of WiFi networks is different in each environment, the WKNN method requires tuning of the parameters, including determination of the most appropriate *k*.

**Figure 4:**
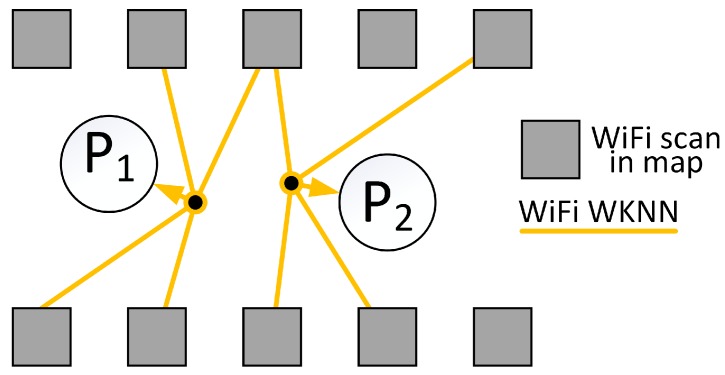
The WiFi fingerprinting measurement is converted into the graph hyperedge that joins the pose of the current scan with the *k* most similar WiFi scans in the map.

In the graph-based optimization, the WiFi WKNN measurement is represented in the form of a hyperedge that joins the place of the WiFi scan with *k* locations of the most similar WiFi scans stored in the map. An exemplary hypergraph is presented in Figure 4. The error of the hyperedge is defined as:

(14) $$e_{f(i)}=\left[\begin{array}{c} x_{i}-\sum_{j=1}^{k}w_{j}\cdot x_{j}^{\mathrm{wifi}}\\ y_{i}-\sum_{j=1}^{k}w_{j}\cdot y_{j}^{\mathrm{wifi}} \end{array}\right],$$

where $\left(x_{i},y_{i}\right)$ is the location where scan was captured and $\left(x_{j}^{\mathrm{wifi}},y_{j}^{\mathrm{wifi}}\right)$ is the location of the *j*-th WiFi scan in the map. In the case of a single user, the location of the *j*-th WiFi scan ($x_{j}^{\mathrm{wifi}},y_{j}^{\mathrm{wifi}}$) is assumed to be fixed (known prior to operation), which is not true in the multi-user scenario. The normalized weight $w_{j}$ of the *j*-th scan is given by:

(15) $$w_{j}=\frac{s(X_{i},Y_{j})}{\sum_{j=1}^{k}s\left(X_{i},Y_{j}\right)},$$

where $s(X_{i},Y_{j})$ is the similarity measure between $X_{i}$ and the *j*-th scan from the map $Y_{j}$.

In the proposed system the similarity measure is set to the inverse of the measured error between the vectors describing two scans and computed as:

(16) $$s\left(X_{i},Y_{i}\right)=\frac{1}{d_{p}\left(X_{i},Y_{i}\right)+\epsilon },$$

where $d_{p}$ is the Euclidean norm between the scans, and the small constant value ϵ is added to prevent division by zero.

When a WiFi measurement is captured (using WKNN), we check if a new node should be added to the factor graph. The decision is taken upon evaluation of the distance from the previous node measured by the PDR. If this distance is too short, the measurement is assumed to be taken from the previous node in the graph. For such a node that has a WiFi observation attached, we change the initial position guess in the graph to the values estimated from the WKNN, as this technique usually provides more accurate user position guess than the incremental PDR system. The new WiFi measurement for a node means that there are two position estimates for this node coming from the PDR and the WiFi/WKNN. Therefore, the optimization procedure is invoked to determine the best estimate of the user pose. In optimization, the Jacobian of the WiFi measurement for the single-user version is given by:

(17) $$J_{f(i)}^{\mathrm{single}}=\left[\begin{array}{cc} \frac{\partial J_{f(i)}^{\mathrm{single}}}{\partial x_{i}} & \frac{\partial J_{f(i)}^{\mathrm{single}}}{\partial y_{i}} \end{array}\right]=\left[\begin{array}{cc} 1 & 0\\ 0 & 1 \end{array}\right]$$

as the WiFi map is fixed and only the location where scan was captured can be optimized. In the multi-user scenario, the location with new scan and locations of WiFi scans captured in other trajectories can be optimized resulting in a more complex Jacobian:

(18) $$J_{f(i)}^{\mathrm{multi}}=\left[\begin{array}{cc} \frac{\partial J_{f(i)}^{\mathrm{multi}}}{\partial x_{i}} & \frac{\partial J_{f(i)}^{\mathrm{multi}}}{\partial y_{i}}\\ \frac{\partial J_{f(i)}^{\mathrm{multi}}}{\partial x_{1}} & \frac{\partial J_{f(i)}^{\mathrm{multi}}}{\partial y_{1}}\\ \ldots & \ldots \\ \frac{\partial J_{f(i)}^{\mathrm{multi}}}{\partial x_{k}} & \frac{\partial J_{f(i)}^{\mathrm{multi}}}{\partial y_{k}} \end{array}\right]=\left[\begin{array}{ccccccc} 1 & 0 & 0 & 0 & \ldots & 0 & 0\\ 0 & 1 & 0 & 0 & \ldots & 0 & 0\\ 0 & 0 & -w_{1} & 0 & \ldots & 0 & 0\\ 0 & 0 & 0 & -w_{1} & \ldots & 0 & 0\\ \vdots & \vdots & \vdots & \vdots & \ddots & \vdots & \vdots \\ 0 & 0 & 0 & 0 & \ldots & -w_{k} & 0\\ 0 & 0 & 0 & 0 & \ldots & 0 & -w_{k} \end{array}\right]$$

In a single- and multi-user scenarios we assume that the locations of the WiFi scans in the initial map are not optimized and the corresponding Jacobians entries are equal to zero.

The strength of the proposed hyperedge is determined by the values in the corresponding information matrix:

(19) $$\Omega_{f}=\left[\begin{array}{cc} k_{\mathrm{wifi}} & 0\\ 0 & k_{\mathrm{wifi}} \end{array}\right],$$

where parameter $k_{\mathrm{wifi}}$ depends on the type of the environment. The parameter can be based on the accuracy of the WKNN WiFi obtained on the testing set during tuning of the $k_{\mathrm{wifi}}$ parameter for the selected environment or just set as a confidence value with respect to the parameters of other types of edges existing in the same graph. In the proposed system, the $k_{\mathrm{wifi}}$ was determined experimentally and was set to 10.

## 5. Multi-User Localization

**Figure 5:**
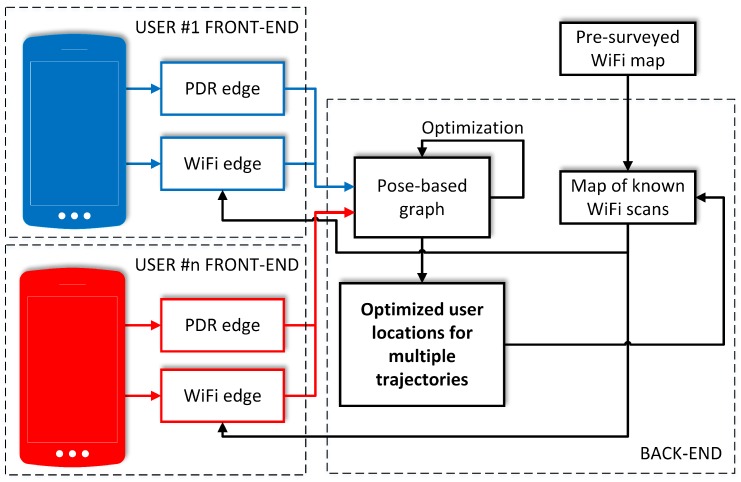
Overview of the processing when a multi-user localization is performed.

Indoor localization systems are usually deployed in sites where multiple users are present. Fortunately, the graph-based formulation of the indoor localization problem can be naturally extended to make it possible to utilize relations between WiFi scans observed by several users. These relations give rise to additional constraints that improve the pose estimation results for the involved users. An overview of the data flow in the multi-user version of our indoor positioning system is shown in Figure 5. While the single-user version was implemented on mobile devices, the multi-user system that has to collect data from many handheld devices requires a central processing unit. Our approach retains the front-end of the single-user system on the mobile devices, but implements a new back-end that runs on a stationary x86 computer connected to the local network. In this version, the back-end is also responsible for keeping the map of WiFi scans that can be discovered by multiple users or predefined in a pre-surveyed map. It should be noticed that while a predefined WiFi map is necessary to pin-point the estimated trajectories to the known environment (by surveying the scans against a blueprint of the floor), this map can be sparse, making the survey quick and easy.

**Figure 6:**
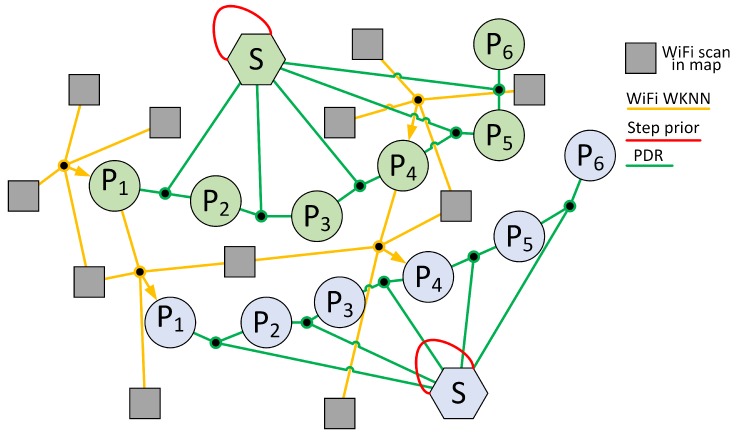
Exemplary multi-user graph created when two trajectories of users (green and blue nodes) are joined by the WiFi WKNN edges.

In the multi-user system, we assume that the WiFi map of the environment is evolving with time. For the first user, we perform typical localization using the PDR with step length estimation and the WiFi WKNN approach. In the case of the second and following users, the WiFi WKNN approach utilizes the stored WiFi map of the environment and the WiFi scans taken by the previous users with their corresponding locations. Contrary to the WiFi scans stored in the predefined map, the estimated locations of WiFi scans taken by users can be changed during the optimization procedure. Therefore, the additional WiFi WKNN edges that join poses from two trajectories make it possible to optimize both user trajectories in the joint optimization problem. An exemplary graph containing trajectories for two users is presented in Figure 6.

The multi-user version of our indoor positioning system does not require any extensions to the mathematical formulation of the localization problem, because the additional constraints between WiFi scans acquired by different users are obtained through the WKNN technique, which is already part of our system. Therefore, the additional edges are established using the hyperedge error function (14) with the respective Jacobians, and the information matrix given by Equation (19).

## 6. Experimental Evaluation

The presented system is focused on utilizing graph-based optimization and multiple user trajectories to increase the accuracy and to make it possible to build a WiFi map with minimal effort. We are not focusing on the performance in the single-user case, as we believe that the proposed approach does not outperform particle filtering with respect to the localization accuracy of an individual user on a dense enough WiFi map. Therefore, the dataset required in our case should contain as many trajectories on a relatively small area as possible. We are unaware of existence of such a dataset, and therefore we have recorded our own sequences, that are made publicly available together with the open-source code of our system.

### 6.1. Experimental Setup

**Figure 7:**
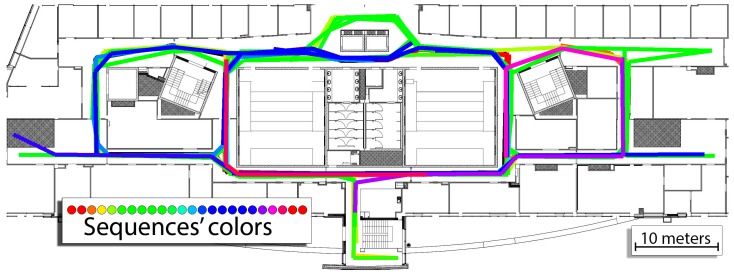
Ground-truth user motion for the 24 recorded sequences as annotated on the map by the users.

The experiments were conducted on the third floor of the Centre of Mechatronics, Biomechanics, and Nanoengineering at Poznan University of Technology. The building has multiple corridors that can be used to access laboratories and lecture halls. In our experiments, we recorded 24 sequences that users would usually make to access these rooms that varied when it comes to the length. Two people were involved in recording the sequences: one responsible for recording 19 sequences and another one that recorded 5 sequences. We used a Sony Xperia Z3 smartphone to capture WiFi, accelerometer and orientation data for further processing. The detailed lengths of the sequences are presented in Table 1. The total distance covered by the sequences according to the ground-truth information is equal to approx. 1565 m. During these sequences, 346 WiFi scans were recorded to support indoor localization. The ground-truth information was obtained from users that annotated the covered paths on the prepared map of the building. The ground-truth routes are presented in Figure 7.

**Figure 8:**
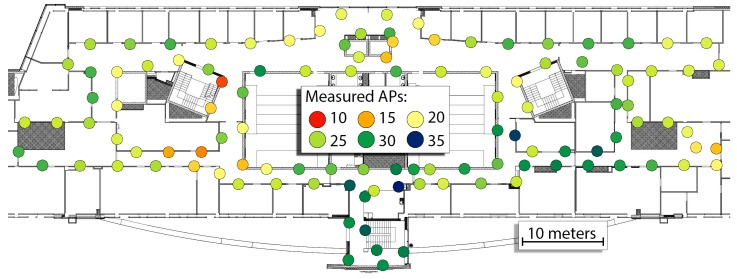
Pre-surveyed WiFi map of the environment highlighting the number of different APs detected in each WiFi scan.

In this research, we assumed that an *a priori* map of WiFi fingerprints is available for the environment. Hence, we recorded WiFi fingerprints along the walls of the corridors. The scans were recorded in-motion along the assumed route with constant walking speed. The dynamic capture procedure was preferred to the static recordings, as it makes possible to quickly capture the required WiFi map, despite being slightly less accurate than the static acquisition approach. The number of APs is based on the number of different measurements in the scans and measurements at 2.4 GHz and 5 GHz from the same APs are treated as independent measurements. The recorded WiFi map consists of 114 scans, and the average number of APs per scan (i.e., user position) is equal to 21.91, but varies per location. The minimum number of APs visible in one location is equal to 10 while the maximum number is equal to 31. The locations with known WiFi scans along with the recorded number of distinct APs in each location are presented in Figure 8.

### 6.2. Tuning the WiFi WKNN System

The performance of the WKNN algorithm depends on the choice of a few parameters that have to be tuned:

- *k* - the number of scans from WiFi map used to estimate the user’s location,
- shared_threshold - the minimal ratio of networks shared between two WiFi scans required to consider them in the WiFi WKNN approach,
- signal_strength_threshold - the minimal signal strength of the measurement that is used in the WiFi WKNN (it is used to remove very weak measurements),
- error_type - the type of error measure (distance norm) used to compare measurements from two scans.

Therefore, we performed a simple exhaustive search of all of the configurations to determine the best values of parameters on recorded sequences. We evaluated the following values of parameters:

- k∈{3,4,5,6,8},
- shared_threshold∈{0.5,0.6,0.7,0.8},
- signal_strength_threshold∈{−100,−90},
- $error\_type\in \{L_{1}\mathrm{norm},L_{2}\mathrm{norm}\}$.

**Figure 9:**
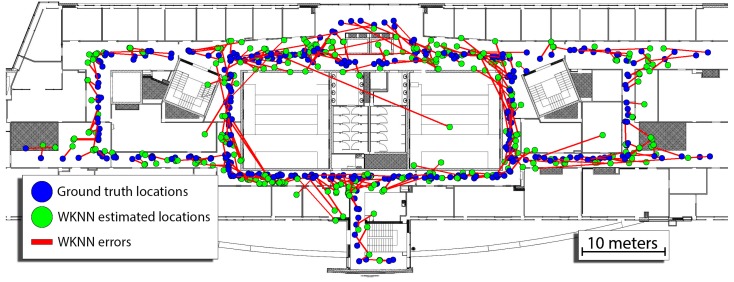
Errors of the WKNN approach reported for the best found configuration of the parameters with ground-truth locations drawn using blue circles, estimated locations marked as green circles and the estimation errors represented as red lines.

The best results were obtained for k=4, shared_threshold=0.6, signal_strength_threshold=−100 and $error\_type=L_{2}$ (Euclidean norm), with a mean error being equal to 2.86 m, third quartile of error equal to 3.66 and the maximum error of 22.47 m. The errors of the WKNN approach, in that case, can be observed in Figure 9. We do not change these parameters in all further evaluations. Probably, more accurate localization could be obtained with a greater *k* parameter when the density of WiFi map is increased, but no clear rules are known to the authors how to make these changes during operation. Therefore, these parameters remain fixed making it possible to clearly show the contribution of the proposed approach.

### 6.3. Localization of a Single User

Firstly, the system is evaluated in single-user scenarios. The proposed graph-based system was evaluated on the 24 recorded sequences. To enable quantitative comparison between the obtained trajectories, a translational trajectory error metric is used, which is similar in concept to the ATE (Absolute Trajectory Error) proposed in Ref. [48]. The ATE measure is a typical tool used in the evaluation of vision-based SLAM systems in robotics when the ground truth is available. The ATE measuring tools cannot be used directly in the case of our dataset, as the ground-truth path G and the estimated trajectory E are not synchronized. This is caused by the fact that an external motion capture system or GPS cannot be used to obtain the ground-truth trajectory over long paths in natural indoor environments. As the result, the ground-truth path is obtained by the users manually marking their route with respect to the walls and objects of known positions in the floor plan.

In the proposed metric, the estimated trajectory E is firstly mapped onto the subsampled ground-truth route G by computing the rigid-body transform S that is the least- square solution to the alignment problem [49]. Then, the trajectory error for the *i*-th pose in the trajectory is computed as:

(20) $$F_{i}=G_{i}^{-1}SE_{i}.$$

Similarly to the ATE, our metric defines the error as the translational component of $F_{i}$ and computes the Root Mean Square Error (RMSE) or the mean error over the whole sequence. Due to the lack of time synchronization, the final error of $F_{i}$ is computed between the given node of E and the closest (in the Euclidean sense) point on the original route G. As a result, the metric error of the trajectory is found while the rotational part is ignored.

**Figure 10:**
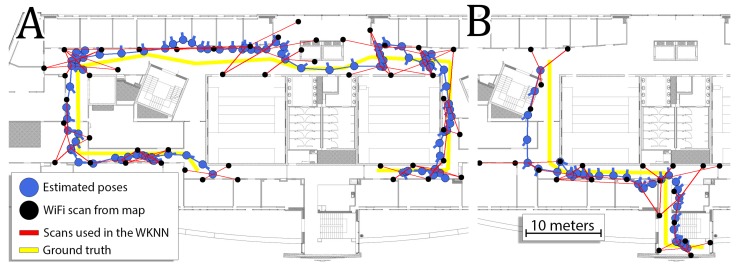
Estimated trajectories compared to the ground-truth paths on sequence 1 (**A**) and sequence 5 (**B**). Constraints imposed by WKNN-based matching of the obtained WiFi scans to the pre-surveyed map are visualized by thin red lines.

The performance of the system on two exemplary sequences is presented in Figure 10. Each blue circle represents one estimated user location that is connected to the next node using a blue edge that represents the PDR estimate. Each black circle represents the location with WiFi scan stored in the map that was utilized at least one time for the WiFi WKNN estimation. The WiFi WKNN estimation is represented by joining the user location with four locations of the scans stored in the map using red lines. The weight of each connection in the WiFi WKNN is not shown in the figure. The ground-truth path is shown using yellow segments.

The system obtains the RMSE of 2.04 m and an average error of 1.29 m on sequence 1 and the RMSE of 3.23 m and an average error of 2.02 m on sequence 5. For all of 24 sequences, the RMSE was measured to be equal to 5.19 m and the average error is equal to 2.21 m. Detailed results are presented in the following section in Table 2. This proves that the proposed processing can be used to estimate the trajectory of the user in the environment. It is important to notice, that the proposed error measure underestimates the real error as the time relations between estimated and ground-truth trajectories are not taken into account. As a result, the delay of the estimate of the user location or incorrect localization along the ground-truth route is not punished in any way. Therefore, the measure should not be used to argue about the absolute accuracy of the system but can be used to compare different configurations of the system and to determine which configuration is more accurate.

### 6.4. Localization of Multiple Users

**Figure 11:**
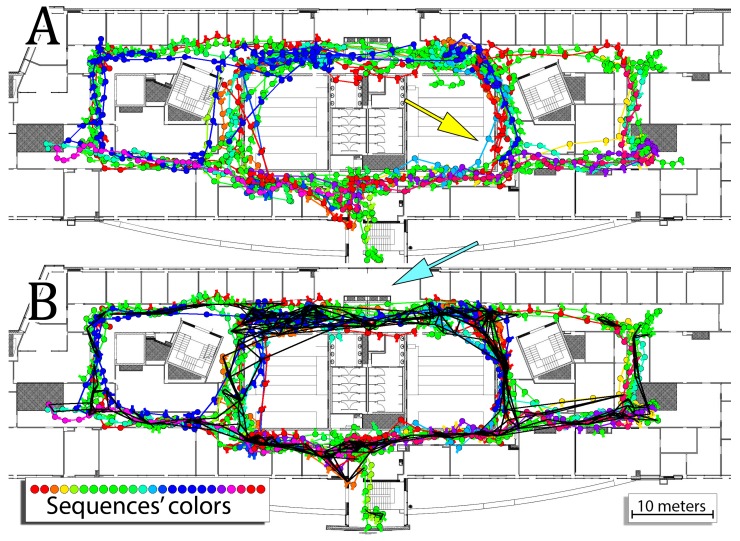
Comparison between trajectories obtained by separate (**A**) and joint (**B**) optimization of factor graphs built for all the 24 trajectories. The black lines in (**B**) represent constraints between poses belonging to trajectories of two different users that emerged by using the WiFi scans collected by the users in the WKNN procedure. Arrows point to areas best illustrating the specific behavior of the system that is explained in the text.

The experimental sequences were recorded in the building along the routes taken by users to reach different destinations. As a result, the same corridors were explored multiple times by different trajectories and all of the sequences were used in the comparison. In the first approach, the graphs for each sequence were constructed and optimized separately and the obtained trajectory estimates were evaluated against the ground-truth information. In the second approach, the relations between different trajectories were utilized. To ease the computation, it was assumed that the trajectories were recorded sequentially meaning that during the processing of the *i*-th sequence it was possible to utilize the information about previous i−1 sequences. In other words, the scan in the currently processed sequence was compared to all scans in the prior WiFi map and all WiFi scans captured by users in the previous sequences. The resulting graph contained edges joining location of users from different trajectories. The whole graph containing information from all sequences is optimized jointly to provide the best possible estimate for all sequences. The results obtained for both configurations of the system are presented in Figure 11 and in Table 2.

When each sequence is optimized separately, the overall RMSE for all sequences is equal to 5.19 and the average error is equal to 2.21. When all of the sequences are jointly optimized with inter-user relations, then the overall RMSE for all sequences in equal to 4.65 whereas the average error is equal to 2.11. Therefore, the performed comparison shows that including relations between multiple trajectories in the graph-based optimization results in more accurate localization estimates.

When considering joint optimization of trajectories, the relations between users occur commonly. In Figure 11, these relations are marked with black lines indicating that the selected WKNN estimation utilized WiFi scan captured on the other trajectory. In the case of the joint optimization, 292 WKNN estimations out of 347 utilize at least one of four selected WiFi scans (*k* = 4 in WKNN) captured in another sequence. It means that 84% of performed WiFi WKNN estimates utilized additional information from other trajectories and is especially high considering that the first trajectory contains 23 WiFi WKNN estimates that can only be based on the original WiFi map. For the 15 out of 24 sequences, all of the performed WKNN estimations used at least one WiFi scan from other sequence meaning that the connections between sequences were critical for the obtained performance of the system.

The positive effect of the introduced relations between trajectories can be observed in Figure 11 for a sequence marked with a light-blue color in a location pointed by the yellow arrow. Including additional information from other users makes it possible to better estimate the motion and thus follow the corridor providing a more accurate estimate of the motion. However, including these additional relations sometimes has a negative influence on the estimation. This effect can be observed for all of the trajectories that follow a path above the elevators on the top of the building blueprint. When sequences are jointly optimized, the optimization tends to bring these sequences to one, more common route located below the elevators, which is marked by the cyan arrow. Therefore, despite the obvious decrease in the estimation error, joint optimization of sequences shows a preference for more commonly used routes and might decrease the accuracy in the case of routes taken with a lower frequency.

**Figure 12:**
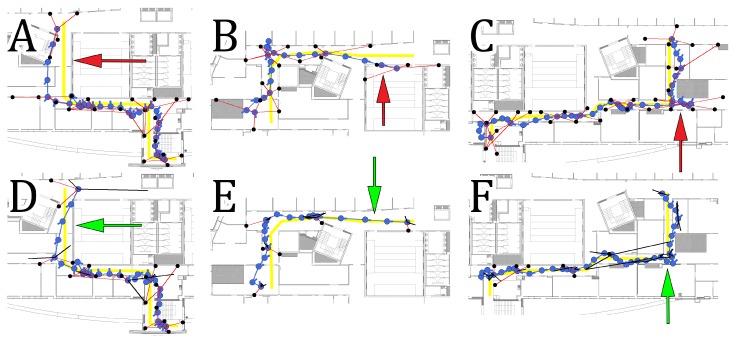
Comparison between trajectories obtained with separate (**A**–**C**) and joint optimization (**D**–**F**) for sequences 5 (**A**,**D**), 18 (**B**,**E**) and 22 (**C**,**F**). Red arrows highlight the inaccurate parts of individually estimated trajectories that were then corrected due to the joint optimization.

A visual comparison between individual trajectories obtained using separate and joint optimization on sequences 5, 18 and 22 can be observed in Figure 12. The performance on the sequence 5 is presented in Figure 12A,D. The lack of proper WiFi measurements and inaccurate orientation changes result in a localization estimate drifting outside of the corridor. This is undesirable as it can be seen clearly that the performance of the system is unsatisfactory for the users. When additional information from other trajectories is used, the WiFi WKNN estimate is more accurate, and the corresponding part of the graph is corrected and the final localization RMSE is reduced from 3.23 m to 1.85 m. A similar situation can be observed for sequence 18 presented in Figure 12B,E. The additional information from another trajectory results in more accurate WiFi WKNN estimation and a final reduction of localization RMSE from 6.54 m to 3.54 m. As a result, the user is no longer localized outside of the corridor and therefore it is possible to appropriately navigate the user to the destination. The last example of trajectories obtained on sequence 22 is presented in Figure 12C,F. The addition of information from other users makes it possible to correct the turn of the user trajectory despite inaccurate orientation estimation that suggested further movement in the same direction. In the case of sequence 22, the RMSE was reduced from 3.68 m to 1.05 m when joint optimization was used.

In the multi-user version of the positioning system, its performance may also depend on the communication efficiency between the handheld devices and the stationary computer hosting the back-end. However, because the communication architecture is out of the scope of this article, all the experiments were performed off-line, on previously saved trajectories to enable a fair comparison between the considered variants of the system. Unfortunately, this approach made it impossible to evaluate the processing time in a realistic way. While we already have demonstrated the ability to optimize a factor graph for the indoor positioning problem on an Android-based smartphone [11], we need to further research this issue for the multi-user case. But it should be noticed that the number of user poses in the considered trajectories varied from 17 to 152, while the number of poses having a WKNN measurement from WiFi scans ranged from 5 to 39—in average only one third of the poses got constraints from the WiFi measurements. Therefore, the number of state variables in the optimized system was considerably smaller than in the mathematically similar visual SLAM problem [40], making it possible to jointly optimize multiple trajectories within a reasonable time.

### 6.5. Performance of the System on the Reduced Original Map

Including the additional relations between trajectories in the optimization system makes it possible to obtain more accurate localization for single users when additional measurements are added to the well-prepared WiFi map that is known prior to operation. But the original WiFi map degenerates over time and needs to be updated, i.e., due to the changes in elements placed in the environment or changes in the broadcasted networks. Therefore, the experiments were conducted to verify how the systems with separate and joint optimization would perform in scenarios with reduced availability of the original WiFi map.

**Figure 13:**
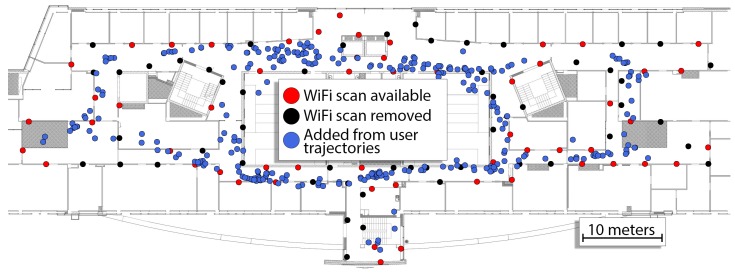
WiFi scans stored in the pre-surveyed map and WiFi scans added from user trajectories. Accurate localization is much harder when only 50% of the pre-surveyed WiFi scans become available in the map (red circles) compared to the full WiFi map (red and black circles). Joint optimization makes it possible to supplement the reduced WiFi map with new scans belonging to other trajectories (blue circles).

To perform these experiments we decided to reduce the size of the original WiFi map to contain from 90% to 50% of the originally stored scans. As the placement of these scans has a huge impact on the accuracy of the WiFi localization, we decided to randomly keep or remove the WiFi scans from the original map in order to achieve the wanted density of the WiFi map. An exemplary map containing half of the original WiFi scans is presented in Figure 13. As this randomness influences the results, we repeated the evaluation procedure 50 times for each configuration. As the optimization converges to a local minimum and is deterministic, the obtained average performance on these 50 trials is a good measure of the performance of the system on a reduced WiFi map. During these experiments, the WKNN parameters were not changed from the originally chosen ones.

**Figure 14:**
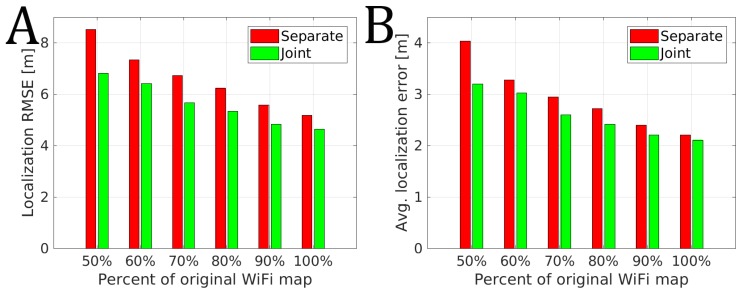
The RMSE (**A**) and average error (**B**) over 50 trials depending on the percent of the size of the original WiFi map when considering trajectories separately and with jointly in the graph-based optimization.

The performance of the system in different configurations is presented in Figure 14. For each configuration, we present the aggregated performance of the system as the average localization RMSE of the system over 24 trajectories and 50 trials. We also consider the mean value of the average localization error over 24 trajectories and 50 trials.

Regardless if we consider sequences separately or jointly in the graph-based optimization, both systems drop in the accuracy when less information from the prior map is provided. This is to be expected as the best performance with the WKNN approach is usually obtained if a dense map of the environment is provided. In the case of the system considering a sequence separately, the drop when 50% of original WiFi scans are available is more significant with the increase of the localization RMSE by 3.34 m when compared to the performance when full WiFi map is available. In the same scenario, joint consideration of trajectories shows a smaller drop in performance with the localization RMSE increased by 2.17 m. The same observation can be made for the average localization error that was larger by 1.83 m for separate trajectories when compared to the increase of 1.09 m for jointly optimized trajectories. When trajectories are jointly optimized only 80% of the original WiFi map is needed to obtain approximately the same accuracy as with 100% of the original WiFi map when sequences are optimized separately. These results suggest that considering relations between multiple trajectories results in a more robust trajectory estimate even in the case when the pre-surveyed WiFi map is degraded significantly.

### 6.6. Step Length Estimation

Many indoor localization systems assume that the step length is known *a priori*, and perform no estimation during the localization. To verify the influence of the step estimation on the obtained user pose estimates, we provided four configurations of our system. In two configurations the trajectories for users were optimized separately and the step length estimation was turned off in one case and turned on in the other one. In the remaining two configurations, the graphs for trajectories were optimized jointly and again the step length estimation was turned off and on, depending on the configuration. Without step length estimation, it was assumed that the step length is equal to 0.65 m, which was also a value assumed as prior when the step length estimation was performed. In each configuration, the step length was estimated separately for each user trajectory and was assumed to be constant through the sequence.

**Figure 15:**
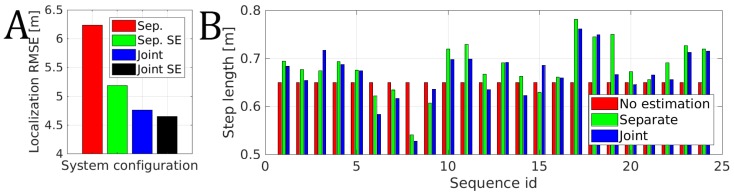
(**A**) The localization RMSE for all sequences depending on the system configuration when considering separate (Sep.) and joint (Joint) optimization without and with (SE) step length estimation. (**B**) The estimated step length depending on the sequence and system’s configuration.

The localization RMSE obtained for all of the recorded sequences is presented in Figure 15A. The worst localization RMSE of 6.24 m was obtained when each trajectory was optimized separately and no step estimation was performed. The same system with step length estimation obtained a localization RMSE of 5.19 m, which is a significant improvement. When the trajectories were optimized jointly, the influence of step length estimation is smaller but visible with a reduction of RMSE from 4.76 m to the RMSE of 4.65 m when the step length estimation is turned on.

The estimated step lengths for different trajectories are presented in Figure 15B. The step length varies significantly changing from 0.53 m to 0.78 m depending on the sequence. This change is related to the walking speed and the height of a person that took these measurements. Unfortunately, we lack the ground-truth information about this parameter and thus can only confirm that a great inaccuracy should be expected when no proper step length estimation is performed.

The 24 sequences were recorded in our case by two people. One person measuring 1.80 m recorded 5 sequences while another measuring 1.93 m was responsible for the remaining 19 sequences. For the shorter person, the average estimated step length was equal to 0.625 m while the standard deviation was equal to 0.065 m depending on the sequence. For the taller person, the average estimated step length was equal to 0.695 m while the standard deviation was equal to 0.038 m. As we lack the proper ground truth, we can only argue that the estimated values are sensible as taller people tend to take larger steps and slightly smaller steps sizes than usually anticipated might be explained by the slower walking speed during the experiment to avoid potential errors.

### 6.7. Comparison to Other Approaches

Unfortunately, the specific requirements of our approach with respect to the recorded user trajectories rendered it impossible to directly evaluate the presented system on any of the publicly available data sets for indoor positioning. However, we can roughly compare the positioning accuracy of our system to the results achieved by several teams participating in the smartphone-based off-line indoor positioning competition held during the Indoor Positioning and Indoor Navigation (IPIN) 2017 conference. The competition and its results are described in Ref. [50]. These results cannot be directly compared to our results shown in Table 2, because of the different environments, a number of available APs, and the methodology of surveying the WiFi map. However, we believe that the advantages of our approach can be assessed qualitatively in comparison to other systems using the data from Ref. [50], because of the similarity of building structures and the similar scale of experiments with respect to the total length of trajectories.

Table 3 summarizes the positioning results in terms of the mean distance to ground-truth error and the third quartile position error for the four systems competing during IPIN 2017 and the FastGraph system [32]. The FastGraph follows a graph-based optimization approach similar to ours and compares also to the results published in Ref. [50], thus the best results of this system (DSI-DEP environment) given in Ref. [32] have been added to the table for comparison. The results for our system are presented for the multi-user version with step length estimation, averaged for all the 24 trajectories. The positioning accuracy of our system is better than the results known from the literature, although unlike the teams competing during IPIN we used only a sparse and rough pre-surveyed WiFi map and we did not calibrate the algorithm with a ground-truth database, such as the one provided for the competing teams. All our experiments used the same sensor models and other parameters (WKNN, step length prior). We achieved results that are at least comparable to those known from the recent literature with a minimized effort of site surveying and parameter tuning. Therefore, we consider our approach a step towards wider acceptance of WiFi-based indoor positioning in practical applications.

## 7. Conclusions

We present an indoor localization system that is based on the graph-based optimization to fuse data from the sensors available in typical mobile devices. The graph-based formulation means that each constraint is represented as an edge in the graph that is later optimized to determine the configuration of nodes that yields the most probable sequence of user locations. The indoor localization system is based on the data coming from a mobile device that are processed to form the PDR and the WiFi WKNN systems. The main novelty of the proposed approach lies in utilizing relations between multiple user trajectories to form global optimization problem that can be solved jointly in order to increase the accuracy and the robustness of the proposed approach. Furthermore, a novel contribution is the additional step length estimation that is a parameter refined during localization for the first time implemented in a graph-based localization system. The contribution is verified on a recorded dataset consisting of 24 sequences with a total length of approx. 1.5 km. As the code of the described system and the used dataset are publicly available, it is possible for everyone to verify our results and build upon our experience.

## Figures and Tables

**Table 1:** Lengths of the recorded sequences. The total length of all recorded sequences is equal to 1565.44 m.

**Sequence**	1	2	3	4	5	6	7	8	9	10	11	12
**Length [m]**	104.4	73.8	81.2	53.3	44.8	159.1	102.7	104.3	112.3	75.4	52.1	112.3
**Sequence**	13	14	15	16	17	18	19	20	21	22	23	24
**Length [m]**	40.7	37.6	65.0	20.4	33.5	37.6	40.2	58.5	43.1	49.5	25.8	37.8

**Table 2:** Comparison between trajectories obtained without (separate) and with (joint) inter-user edges on all available sequences. The values are bolded for cases when joint optimization decreased the localization RMSE by over 20% and the final error was below 4 m, which was assumed to be acceptable for navigational purposes.

Localization Error of Separately Optimized Trajectories						Localization Error of Jointly Optimized Trajectories					
Seq.	**RMSE**	**Avg. Err.**	Seq.	**RMSE**	**Avg. Err.**	Seq.	**RMSE**	**Avg. Err.**	Seq.	**RMSE**	**Avg. Err.**
1	2.04	1.29	13	2.53	1.84	1	2.02	1.31	13	3.1	1.76
2	1.42	0.85	14	5.23	2.67	2	**1.08**	0.68	14	4.92	2.91
3	7.13	3.24	15	1.94	1.15	3	7	3.04	15	1.83	0.88
4	4.19	2.52	16	0.89	0.64	4	3.8	2.1	16	1.14	0.87
5	3.23	2.02	17	4.31	2.15	5	**1.85**	1.19	17	6.52	4.07
6	2.54	1.46	18	6.54	3.95	6	2.67	1.38	18	**3.54**	1.88
7	1.83	1.11	19	2.82	1.88	7	**1.23**	0.8	19	2.69	1.6
8	4.38	2.04	20	2.93	1.36	8	5.36	2.53	20	3.34	1.45
9	6.04	2.87	21	1.66	1.14	9	5.49	2.93	21	1.63	1.07
10	14.04	7.21	22	3.69	1.76	10	10.67	5.75	22	**1.05**	0.6
11	3.18	2.16	23	5.95	4.27	11	4.13	2.31	23	7.38	4.82
12	2.28	1.31	24	9.98	4.62	12	4.39	2.47	24	7.05	3.76
All	5.19	2.21				All	4.65	2.11			

**Table 3:** Comparison of the positioning accuracy of our system in its final configuration to results known from the literature: four teams in the 2017 IPIN competition [50] and the FastGraph system [32].

Positioning System or Team Name	Mean Error [m]	Third Quartile Error [m]
UMinho Team	3.00	3.48
AraraDS Team	3.74	3.53
Yai Team	3.51	4.41
HFTS Team	3.52	4.45
FastGraph (DSI-DEP)	5.08	6.21
our system (multi-user)	2.11	2.12
